# PICNIC web server for predicting proteins involved in biomolecular condensates

**DOI:** 10.1093/bioinformatics/btaf647

**Published:** 2025-12-01

**Authors:** Anna Hadarovich, Maxim Scheremetjew, Hari Raj Singh, HongKee Moon, Lena Hersemann, Agnes Toth-Petroczy

**Affiliations:** Max Planck Institute of Molecular Cell Biology and Genetics, Pfotenhauerstaße 108, 01307 Dresden, Germany; Center for Systems Biology Dresden, Pfotenhauerstraße 108, 01307 Dresden, Germany; Max Planck Institute of Molecular Cell Biology and Genetics, Pfotenhauerstaße 108, 01307 Dresden, Germany; Center for Systems Biology Dresden, Pfotenhauerstraße 108, 01307 Dresden, Germany; Max Planck Institute of Molecular Cell Biology and Genetics, Pfotenhauerstaße 108, 01307 Dresden, Germany; Max Planck Institute of Molecular Cell Biology and Genetics, Pfotenhauerstaße 108, 01307 Dresden, Germany; Center for Systems Biology Dresden, Pfotenhauerstraße 108, 01307 Dresden, Germany; Max Planck Institute of Molecular Cell Biology and Genetics, Pfotenhauerstaße 108, 01307 Dresden, Germany; Center for Systems Biology Dresden, Pfotenhauerstraße 108, 01307 Dresden, Germany; Max Planck Institute of Molecular Cell Biology and Genetics, Pfotenhauerstaße 108, 01307 Dresden, Germany; Center for Systems Biology Dresden, Pfotenhauerstraße 108, 01307 Dresden, Germany; Cluster of Excellence Physics of Life, TU Dresden, 01062 Dresden, Germany

## Abstract

**Motivation:**

Biomolecular condensates have been implicated in key cellular processes such as gene regulation, stress response, and signaling, and dysregulation of condensates has been linked to neurodegeneration and other diseases. Computational algorithms that predict protein condensation can aid systematic characterization of biomolecular condensates at the proteome scale. However, many experimental labs may lack the computational background or resources to run sophisticated prediction tools locally.

**Results:**

Here, we developed the web server implementation of the PICNIC (Proteins Involved in CoNdensates In Cells) machine learning algorithm. PICNIC uses sequence- and structure-based features derived from AlphaFold2 models to predict if a protein is involved in biomolecular condensates. In case of well-studied proteins with available annotations, the user can further benefit from an extended model, PICNIC-GO, which includes additional features based on Gene Ontology terms. Benchmark tests show that PICNIC algorithms predict condensate forming proteins with ∼80% accuracy. By providing an easy-to-use web server, researchers, without specialized expertise, can rapidly test hypotheses about any protein of interest, including designed and mutated sequences.

**Availability and implementation:**

The PICNIC webserver is available at https://picnic-bio.org/.

## 1 Introduction

Protein condensation is an essential mechanism underpinning various cellular functions, including gene regulation, stress response, and signaling (Banani *et al.* 2017, Lyon *et al.* 2021). The formation of biomolecular condensates (condensates for short) orchestrates the spatial and temporal control of biological processes, while their dysregulation has been associated with neurodegenerative disorders and other pathologies (Patel *et al.* 2015, Alberti and Dormann 2019, Alberti and Hyman 2021). Understanding the determinants and consequences of protein condensation is thus critical for unraveling fundamental cellular biology and disease mechanisms. There are more than a hundred unique biomolecular condensates known to date as catalogued by the CD-CODE database and their number is continuously rising (cd-code.org) (Rostam *et al.* 2023). It is important to note that not all condensates form via liquid–liquid phase separation and not all have liquid-like properties. The term biomolecular condensates refer to all non-stochiometric macromolecular assemblies in cells which form via phase transitions. In terms of material properties, liquid-like (Brangwynne *et al.* 2009), gel-like (Law *et al.* 2023), glass-like (Jawerth *et al.* 2020), and solid-like (Patel *et al.* 2015) condensates have been described in the literature. Condensates can also assemble on lipid bilayer surfaces, where they are either anchored by transmembrane proteins or associated peripherally with the membrane (Kim *et al.* 2024). In contrast to non-stoichiometric condensates, stoichiometric, stable molecular assemblies like the ribosome are not considered condensates. We also distinguish between biomolecular condensates (i.e. condensates observed *in vivo* or *in cellulo*) and synthetic condensates that are observed *in vitro* often containing only few purified components.

Most biomolecular condensates contain hundreds to over a thousand different types of proteins as well as nucleic acids and small molecules. Large-scale experimental characterization of condensates *in vivo* is challenging. Therefore, computational methods can facilitate the process of characterizing proteins involved in biomolecular condensates at proteome-scale and across organisms (Hadarovich *et al.* 2025).

PICNIC (Proteins Involved in CoNdensates In Cells) is a machine learning algorithm that predicts proteins involved in biomolecular condensates (Hadarovich *et al.* 2024). We trained two models: PICNIC utilizes sequence- and structure-based features derived from AlphaFold2 models; and an extended model includes additional features based on Gene Ontology terms (PICNIC-GO). Specifically, the models integrate four main types of features: (i) intrinsic disorder scores from IUPred and sequence complexity measures to capture low-complexity regions; (ii) short- and long-range amino acid co-occurrence patterns in the primary sequence; (iii) structure-based features from AlphaFold2 models, combining secondary structure (STRIDE) and residue-level confidence scores (pLDDT) into amino acid–structure–confidence triads; and (iv) for PICNIC-GO, selected high-frequency Gene Ontology terms for molecular function and biological process. Together, these features represent both sequence-intrinsic properties and, where available, functional annotations relevant to condensate formation. PICNIC was trained on experimentally observed human condensate forming proteins in CD-CODE (positive dataset) (Rostam *et al.* 2023) and an unbiased negative dataset consisting of proteins that do not interact with known condensate forming proteins based on human protein-protein interaction network.

In terms of machine learning architecture, both PICNIC and PICNIC-GO are gradient boosting-based models and use ensembles of 10 CatBoost classifiers with early stopping. Starting from 2467 (PICNIC) and 3469 (PICNIC-GO) initial features, we applied iterative feature selection based on feature importance, yielding final sets of 92 and 18 features, respectively. The smaller feature set in PICNIC-GO reflects that GO terms already encode aspects of condensate-related sequence and structural properties. PICNIC was designed to predict condensate forming proteins irrespective of their structural disorder content, and it can successfully identify not only disorder containing but fully ordered proteins that form/localize to condensates (Hadarovich *et al.* 2024).

## 2 Methods

**Figure 1.**
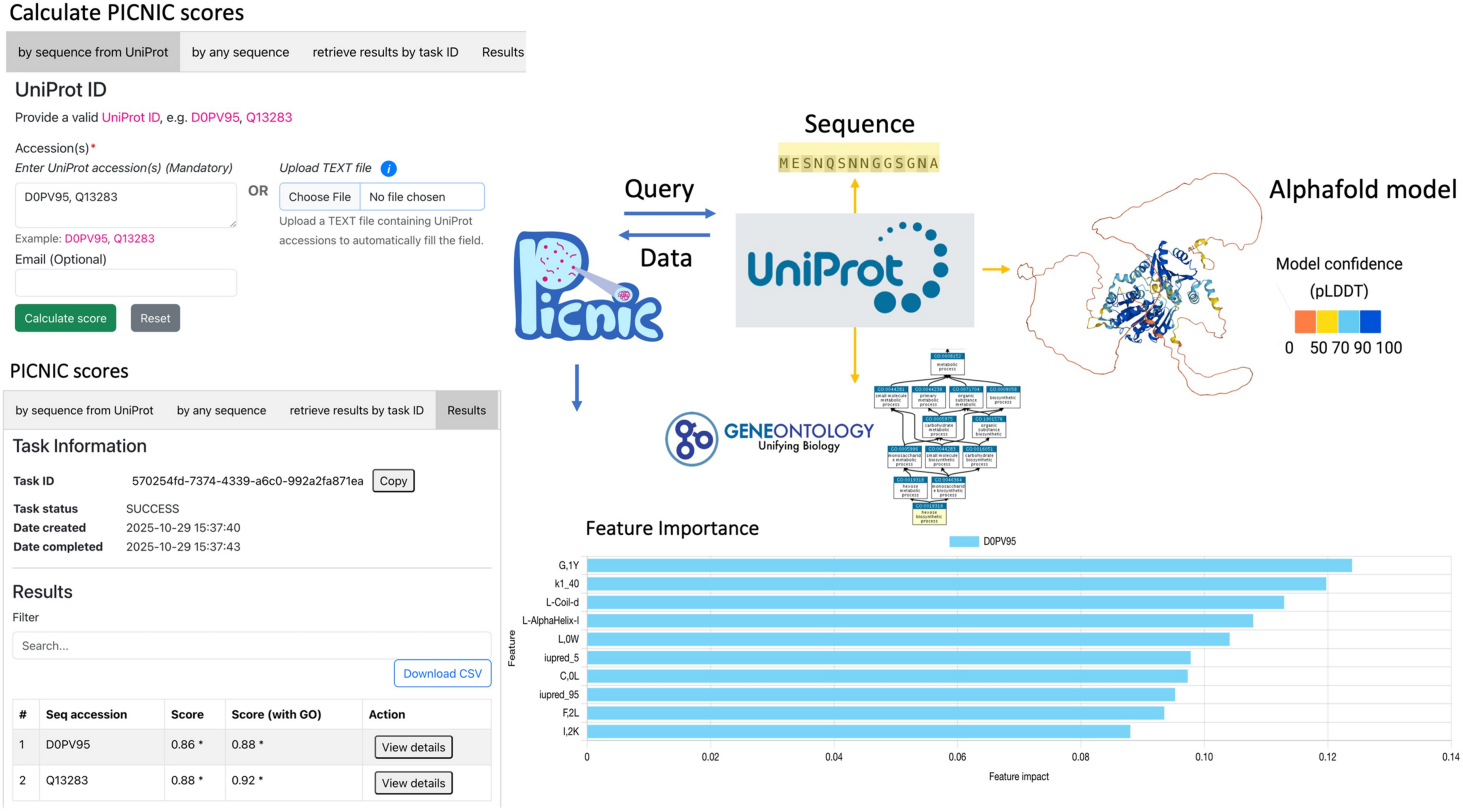
Workflow of the PICNIC web server. The user can input either the UniProt ID of a protein or multiple proteins in the input field or upload a text file with specified UniProt IDs (“by sequence from UniProt” tab). It is possible to calculate PICNIC score for any protein of interest by provinding the sequence in FASTA format (“by any sequence” tab). The AlphaFold2 model in PDB format and Gene Ontology terms in JSON format are retrieved automatically from UniProtKB. The PICNIC model uses sequence and structural information for the score calculation, while the PICNIC-GO model includes Gene Ontology terms as well for score calculation. In case of designed or synthetic sequences (or sequences not available in UniProtKB), the user has to upload the AlphaFold2 structure file (PDB format). In the absence of sequence and/or AlphaFold2 PDB file the job fails (Task status is “FAILURE” in the Results tab). If the calculation is successful, the results are displayed and the feature importance graph is shown to aid interpretation.

The PICNIC web server takes an UniProt ID or AlphaFold2 model of a protein as input and runs both PICNIC and PICNIC-GO algorithms. The model outputs the probability of a protein being a condensate member calculated with PICNIC (PICNIC score) and PICNIC-GO (PICNIC-GO score) algorithms respectively, with scores ≥0.5 indicative of predicted protein condensation. To interpret the PICNIC score, the individual protein features which contribute to the condensation prediction are visualized (Fig. 1). PICNIC was trained on a large positive and negative dataset for the prediction of proteins involved in condensates in vivo. On a test set of 338 positive and 299 negative examples, i.e. proteins that were not part of the PICNIC training set, PICNIC achieved superior F1-score = 0.81 in comparison with other models such as Pdps-8fea (Chen *et al.* 2022) (F1-score = 0.78), PSAP (van Mierlo *et al.* 2021) (F1-score = 0.75) and DeePhase (Saar *et al.* 2021) (F1-score = 0.69) (Table 1). Among the predictors using additional experimental data, PICNIC-GO provides best performance, with an F1-score of 0.84. The performance was similar on two additional datasets, the OpenCell nuclear puncta and PhaSepDB datasets (Table 1). Experimental validation of 21 out 24 positive predictions (87.5%) were correct (formed mesoscale foci) (Hadarovich *et al.* 2024). Although PICNIC was trained on human data, it generalizes well to proteomes of other organisms and it correctly identifies members of biomolecular condensates: 74% for *Mus musculus* (*N* = 1644), 84% for *Arabidopsis thaliana* (*N* = 1497), 87% *Saccharomyces cerevisiae* (*N* = 583) (Hadarovich *et al.* 2024).

**Table 1.** Benchmark results of PICNIC on three different test datasets.^a^

	CD-CODE test set		OpenCell test set		PhaSepDB test set	
Method	AUC	F1-score	AUC	F1-score	AUC	F1-score
**PICNIC**	**0.86**	**0.81**	**0.76**	**0.21**	**0.80**	**0.50**
PSAP	0.77	0.75	0.61	0.14	0.62	0.34
PdPs-8fea	0.83	0.78	0.67	0.15	0.69	0.39
DeePhase	0.69	0.69	0.52	0.09	0.55	0.31

a Test dataset from PhaSepDB (You *et al.* 2020) high-throughput has 441 positive and 1998 negative examples, the test dataset from OpenCell (Cho *et al.* 2022) has 78 positive and 1998 negative examples, and the test dataset from CD-CODE (Rostam *et al.* 2023) is balanced and has 338 positive and 299 negative examples.

PICNIC web is free and open to all users and there is no login required. We use a common design concept for the underlying backend architecture, which allows the execution of computationally expensive jobs of various sizes asynchronously. This has the advantage that the website will not be blocked until a job has finished. The Python-based Django web framework (https://www.djangoproject.com/) is used as the backbone for receiving requests from the frontend client. Gunicorn (https://gunicorn.org/) acts as a WSGI HTTP webserver between the frontend client (e.g. a browser) and Django using the WSGI protocol for the communication. Requests are processed by Django, which means they get validated, approved or declined and transformed into new Celery tasks/jobs. Once a new job has been created, Django will submit the task to a job queue. We use Celery (https://docs.celeryq.dev/en/stable/index.html) for managing the task execution. For the communication between Django and the distributed task queue manager, we use RabbitMQ as a messenger (https://www.rabbitmq.com/).

Jobs scheduled in the queue are picked up by pre-configured workers. Any worker can run a single job at any time. The number of workers is configurable depending on the computing power of the server where the application is running. This allows horizontal scaling if needed. The results of a finished task are stored in the backend database and the status of the job is communicated back to the Django application. The front-end client monitors the status of a job by using the HTTP GET protocol.

We utilized the Vue.js framework (https://vuejs.org/) to create the front-end pages for our web application. Additionally, we used vue-chartjs (https://vue-chartjs.org/) for the feature importance bar chart on the result page.

Each service (Django, Postgres database, messenger, etc.) is implemented as a Docker container and communicates with other services using specific protocols and networks. All services are containerized using Docker Compose, enabling multi-container deployment for scalability and easy reproducibility.

## 3 Usage

**Figure 2.**
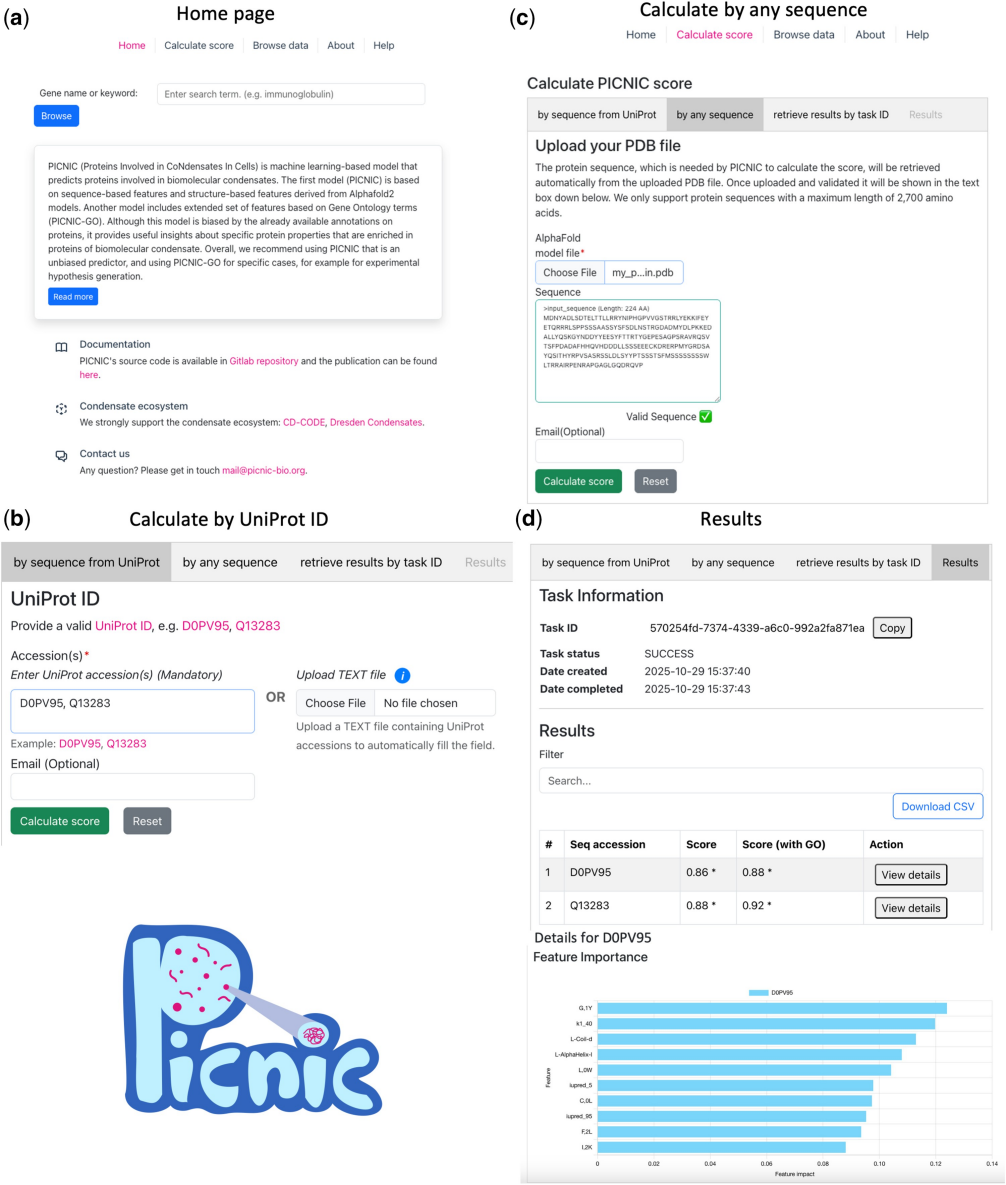
The PICNIC web application provides a user-friendly front end. (a) Home page of PICNIC web server. (b) PICNIC scores for the protein with the known UniProt ID (in case there is an available AlphaFold model in UniProt) can be calculated using the tab “by sequence from UniProt” (Search page). An example is shown for the protein with UniProt IDs “D0PV95” and “Q13283.” The web-service allows to input a UniProt IDs and email (for the notification of the result, optional). (c) Search tab allows to calculate PICNIC scores for any protein sequence (tab “by any sequence”) given the corresponding AlphaFold model (provided by the user). In this scenario the protein sequence is retrieved from the AlphaFold model file. (d) “Results” tab shows the Task status and calculated PICNIC and PICNIC-GO scores (in case of “SUCCESS” Task status). Each job is assigned a Task ID, and the results can be retrieved for 90 days by searching by Task ID in the tab “retrieve results by task ID.” “View details” button in Action tab allows to show the feature importance plot with the impact of the top 10 features which contribute the most to the condensation of a given protein.

The PICNIC web server can be applied to any protein sequence of interest (Fig. 1). In case the protein is present in UniProt and has an associated AlphaFold2 structure in UniProt, the user can submit a calculation by providing the UniProt ID (i.e. six-letter identifier) and (optionally) input an email address to retrieve the results. If an email is provided, the user will receive a notification about the results once the calculation has finished. The results will be stored for 90 days and can be accessed and searched by the Task ID (which is unique and is assigned to the calculation once the job is created) (Fig. 2). Once the UniProt IDs are submitted (via the input field or by uploading a text file), the server creates a request to UniProtKB to download the sequences in FASTA format, AlphaFold2 models in PDB format, Gene Ontology terms in JSON format (Ashburner *et al.* 2000, Jumper *et al.* 2021, UniProt Consortium 2021, Varadi *et al.* 2022, 2024). The PICNIC model uses this information for the score calculation and shows the result of the calculation. It is worth to mention, that Gene Ontology terms are only used for PICNIC-GO score calculation. If the calculation was successful, the Task Status is “SUCCESS,” otherwise: “FAILURE.” The results can be viewed later on the results tab in a table view, that can be searched by the corresponding Task ID. The result table is searchable and downloadable. More details can be accessed by clicking on the “View details” button in the Action tab, which displays the feature importance plots with the impact of the top 10 features which contribute the most to the condensation of a given protein. Upon request, an email is sent to the user once the calculation job finished. The email body contains the UniProt ID and both PICNIC scores as well as a link to the result page. A PICNIC (or PICNIC-GO) score of ≥0.5 means, that this protein is predicted to be a member of biomolecular condensate.

Moreover, PICNIC score can be calculated for any protein sequence, including mutants of existing proteins or any synthetic and designed protein sequences. In this case, the user has to provide an AlphaFold2 or AlphaFold3 model as a PDB file. If not found in AlphaFold database (https://alphafold.ebi.ac.uk/), it can be pre-computed, e.g. using Colab notebook: https://colab.research.google.com/github/sokrypton/ColabFold/blob/main/AlphaFold2.ipynb, where one needs to paste the protein sequence and run the cells in the notebook (Mirdita *et al.* 2022, Varadi *et al.* 2024). The structures retrieved from AlphaFold3 server (https://alphafoldserver.com/) (Abramson *et al.* 2024) can also be used, but they need to be converted from cif format to pdb format, e.g. using Pymol tool (Schrödinger 2015). In this case no protein sequence in FASTA format is needed, because the sequence is retrieved from the PDB file (Fig. 2c). The workflow for the calculation is the same as for proteins defined by Uniprot ID, except that no PICNIC-GO score is provided, since no GO annotations are available. The result page shows scores together with the bar chart (Fig. 2d) representing the most important features contributed to the condensation of a given protein.

## 4 Conclusions

In summary, we present a webserver to compute PICNIC scores for any protein sequence of interest given AlphaFold model, that is either automatically fetched from UniProt database or can be uploaded by the user in case of using new, mutated or synthetic sequences. The webserver outputs the score and visualizes the most important features contributing to the condensate prediction of a given protein for easy interpretation (Fig. 2d).

While several computational predictors of condensate proteins exist, PICNIC addresses key limitations by remaining unbiased with respect to disorder content, generalizing effectively across diverse organisms, and generating predictions for any protein sequence of interest. The availability of a webserver will allow researchers across diverse fields to derive hypotheses about proteins sequences contributing to biomolecular condensate formation.

## Data Availability

All data underlying the algorithm development, validation, bechmarking is available as part of (REFERENCE to Hadarovich et al. 2024) and the precomputed scores are available at https://picnic-bio.org. The codebase of the PICNIC algorithms can be found at https://doi.org/10.5281/zenodo.17877147 or https://git.mpi-cbg.de/tothpetroczylab/picnic/.
